# Whole-Genome Sequencing to Identify Mutants and Polymorphisms in *Chlamydomonas reinhardtii*

**DOI:** 10.1534/g3.111.000919

**Published:** 2012-01-01

**Authors:** Susan K. Dutcher, Linya Li, Huawen Lin, Leslie Meyer, Thomas H. Giddings, Alan L. Kwan, Brian L. Lewis

**Affiliations:** * Department of Genetics, Washington University School of Medicine, St. Louis, Missouri 63110; † MCD Biology, University of Colorado, Boulder, Colorado 80309; ‡ Department of Computer Science and Engineering, Washington University, St. Louis, Missouri 63130

**Keywords:** intraflagellar transport (IFT), flagellar assembly, mating-type, basal bodies, mapping

## Abstract

Whole-genome sequencing (WGS) provides a new platform for the identification of mutations that produce a mutant phenotype. We used Illumina sequencing to identify the mutational profile of three *Chlamydomonas reinhardtii* mutant strains. The three strains have more than 38,000 changes from the reference genome. NG6 is aflagellate and maps to 269 kb with only one nonsynonymous change; the V_12_E mutation falls in the *FLA8* gene. Evidence that NG6 is a *fla8* allele comes from swimming revertants that are either true or pseudorevertants. NG30 is aflagellate and maps to 458 kb that has six nonsynonomous changes. Evidence that NG30 has a causative nonsense allele in *IFT80* comes from rescue of the nonswimming phenotype with a fragment bearing only this gene. This gene has been implicated in Jeune asphyxiating thoracic dystrophy. Electron microscopy of *ift80-1* (NG30) shows a novel basal body phenotype. A bar or cap is observed over the distal end of the transition zone, which may be an intermediate in preparing the basal body for flagellar assembly. In the acetate-requiring mutant *ac17*, we failed to find a nonsynonymous change in the 676 kb mapped region, which is incompletely assembled. In these strains, 43% of the changes occur on two of the 17 chromosomes. The excess on chromosome 6 surrounds the mating-type locus, which has numerous rearrangements and suppressed recombination, and the changes extend beyond the mating-type locus. Unexpectedly, chromosome 16 shows an unexplained excess of single nucleotide polymorphisms and indels. Overall, WGS in combination with limited mapping allows fast and accurate identification of point mutations in *Chlamydomonas*.

Analysis of how organisms develop and function has been greatly aided by the use of forward genetic analysis to screen for mutant organisms in which a specific biological process is disrupted. One of the drawbacks of forward genetic screens is the time and effort needed to find the mutated gene by the use of meiotic mapping to identify the mutant locus. Whole-genome sequencing (WGS) provides an alternative to a map-based approach by directly identifying the molecular lesion in a mutated strain isolated from a genetic screen (Doitsidou *et al.* 2010; Hobert 2010; Sarin *et al.* 2010) as used in *Caenorhabditis* *elegans* (Doitsidou *et al.* 2010; Hobert 2010; Sarin *et al.* 2010), *Drosophila melanogaster* (Blumenstiel *et al.* 2009; Wang *et al.* 2010), and *Schizosaccharomyces pombe* (Irvine *et al.* 2009). Given the small genome sizes of many model organisms, it is possible to easily obtain sufficient coverage that is necessary for the identification of mutants in organisms with respect to a reference genome.

*Chlamydomonas reinhardtii*, a unicellular, biflagellate photosynthetic alga, has provided important insights into cilia/flagella assembly and function, chloroplast function and assembly, and phototransduction (Harris 2009). Large collections of chemically and irradiation induced mutations exist, but the identification of their cognate genes by positional cloning has been slow (Dutcher *et al.* 2002; Dutcher and Trabuco 1998; Hendrickson *et al.* 2004; Iomini *et al.* 2009a; McCarthy *et al.* 2004; Mueller *et al.* 2005). Because flagella from *Chlamydomonas* are amazingly similar to the cilia and flagella from mammals (Li *et al.* 2004) despite the 10^9^ years of evolution separating them, we wanted to be able to identify additional genes responsible for many of the identified flagellar mutant phenotypes.

Previous analysis of two *Chlamydomonas* mutants lacking flagella by electron microscopy showed defects in closing the B-tubule of the outer microtubules (NG6) and extra transition zone material (NG30) (McVittie 1972). The failure to close the B-tubule is observed in the *henin* mouse mutant, which is a defect in Arl13, a small GTPase (Caspary *et al.* 2007; Horner and Caspary 2011). In addition, the zebrafish *fleer* mutant (Pathak *et al.* 2007), and *Drosophila* with mutations in glutamates in the C-terminus of β2-tubulin (Hoyle *et al.* 2008) show a failure in the formation or closure of the B tubule. Both of these defects result from the requirement to posttranslationally modify tubulin by polyglutamylation and/or polyglycylation. Furthermore, knockdown of a tubulin polyglutamylase in zebrafish demonstrated effects on tubulin polyglutamylation, cilia formation, and motility similar to *fleer* mutants (Pathak and Drummond 2009). Extra transition zone material has been observed in the *uni1* (Huang *et al.* 1982) and *uni2* (Piasecki *et al.* 2008; Piasecki and Silflow 2009) mutants in *Chlamydomonas*. These two long-standing unmapped mutant strains from McVittie provide an opportunity to use WGS via massively parallel sequencing technologies to identify the mutations responsible for these phenotypes and to determine the extent of polymorphism in these strains compared with the reference genome.

## Materials and Methods

### Strains and culture media

Strains S1D2 (CC-1952), NG6 (CC-829), NG30 (CC-916), and *ac17* (CC-951) were obtained from the *Chlamydomonas* Genetics Center. Each strain was backcrossed to either 137*mt+* (CC-125) or 137*mt−* (CC-124) strain to remove any unlinked modifiers multiple times. Cells were grown as previously described (Holmes and Dutcher 1989). Crosses, maturation of zygotes, and tetrad analysis were performed as previously described (Dutcher 1995).

### Linkage analysis

To determine linkage, mapping was performed in meiotic progeny from a cross to CC-1952 (Gross *et al.* 1988). Markers for placement onto a particular chromosome were identified in previous work (Bowers *et al.* 2003; Iomini *et al.* 2009b; Rymarquis *et al.* 2005). New markers on a chromosome with linkage were designed *de novo* (supporting information, Table S1).

### *Chlamydomonas* genomic DNA preparation and Sanger sequencing

A DNA mini-prep protocol was used (Lin *et al.* 2010). Sequencing was performed by PNACL (Washington University, St. Louis, MO) and analyzed with Sequencer software (Gene Codes Co., Ann Arbor, MI).

### *Chlamydomonas* genomic DNA preparation for DNA-Seq

A DNA preparation protocol was modified from Lin *et al.* (2010). Approximately 5 × 10^8^ cells were washed in 1 mL of 1× TEN (150 mM NaCl; 10 mM EDTA pH 8.0; 10 mM Tris-HCl, pH 8.0) and resuspended in 300 µL of chilled water. A total of 600 µL of sodium dodecyl sulfate (SDS)-EB buffer (2% SDS; 100 mM Tris-HCl, pH 8.0; 400 mM NaCl; 40 mM EDTA, pH 8.0) and 100 µL of 20% SDS was added to the cells, and the samples were incubated at room temperature for 15 min to ensure complete cell lysis. DNA was extracted once with 1 mL of phenol/chloroform (1:1) and followed by another extraction with 1 ml chloroform. DNA was precipitated with 1 mL of isopropanol on ice for 15 min and centrifuged at room temperature for 10 min followed by a wash with 70% ethanol. DNA was dried with the use of Savant SpeedVac (Thermo Scientific, Waltham, MA) and resuspended in 400 µL of TE (10 mM Tris–HCl, pH 8.0; 1 mM EDTA, pH 8.0) with 5 µg/mL RNase A. The samples were incubated at 37° for 1 hr. DNA extraction with equal volume of phenol/chloroform and chloroform was repeated, followed by precipitation with 1/10 volume of 3 M NaOAc (pH 5.2) and equal volume of isoproponal. DNA was washed with 70% ethanol before SpeedVac dry. A total of 40 µL of TE was used to resuspended DNA. The concentration of DNA was determined by spectrophotometry at 260 nm (Biophotometer 6131; Eppendorf, Westbury, NY). The final concentration of each sample was approximately 200 ng/µL with a 260/280 ratio of 1.80~1.95. Then, 3 µg of *Chlamydomonas* genomic DNA was submitted to Cofactor Genomics or Genome Technology Access Core for library construction and Illumina sequencing.

### Computational analysis of Illumina reads

The 36-bp single-end Illumina sequencing of NG6 was performed by Cofactor Genomics (St. Louis, MO) on the GA-II platform from Illumina (San Diego, CA) and the 101-bp paired-end Illumina sequencing of NG30, and *ac17* was performed by the Genome Technology Access Core (Department of Genetics, Washington University) on the HiSeq platform from Illumina (San Diego, CA). Sequence reads were aligned onto the JGI version 4 *Chlamydomonas* genome assembly with Novoalign (novocraft.com; *options*: -r random -l 25 -e 100 -H -c 12). Sites of single nucleotide polymorphisms (SNPs) in mutant strain genome sequences were computed by use of the pileup (*options*: -v -c -T 0.9) and varFilter (*options*: -D 99999) methods that are part of the SAMtools software suite (Li *et al.* 2009). Genomic SNPs and indels with less than 10 reads were excluded from further analysis. Nonsynonymous and synonymous changes that occur within the predicted coding regions were manually analyzed.

### Electron microscopy

Cell pellets were prepared for electron microscopy by high-pressure freezing followed by freeze substitution as described (Preble *et al.* 2001; O’Toole *et al.* 2003). Serial thin sections, of 50−60 nm were viewed in a Philips CM10 electron microscope operating at 80 kV or 100 kV. Using the microscope’s goniometer stage, the sections were tilted to an angle that permitted the basal body microtubules to be viewed in cross section (Fromherz *et al.* 2004).

### Other methods

*Chlamydomonas* BAC DNA was prepared using QIAGEN Plasmid Midiprep kit as described previously (Dutcher *et al.* 2002). Ultraviolet mutagenesis was performed on cells grown on solid R medium provided for 3 days until cells reached a density of ∼10^8^. The cells were subjected to UV irradiation at 70 mJ (UV Stratalinker 1800; Stratagene, Cedar Creek, TX) and recovered in the dark overnight. The plate was divided into 13 sections and cells were scraped off the plate and spread on 48 25 × 150 tubes with 20 mL of R medium. The top 5 mL was transferred 5 times to enrich for swimming cells.

## Results

The NG6 and NG30 strains lacked flagella and were each crossed to a wild-type parent (CC-125), and immotile progeny from the cross were mated three successive times to CC-125 because the first round of crosses had poor viability (19% and 22%, respectively). After four rounds of crosses, the aflagellate phenotype segregates in a 2:2 pattern in 140 and 170 tetrads, respectively, with excellent viability (>90% with four viable progeny). We then mapped each phenotype by using dCAPs markers in progeny from a cross to CC-1952 to a chromosome (Bowers *et al.* 2003) and then designed new chromosome-specific markers to narrow the region of interest (see Table S1 for primers and conditions used). NG6 maps to chromosome 12 in a region of ∼ 269 kb between markers 160146 and 269006 based on 184 meiotic progeny. This region has 43 genes predicted by GreenGenie2 (Kwan *et al.* 2009) and 49 predicted genes by JGI (Merchant *et al.* 2007). NG30 maps to chromosome 3 to a region of 458 kb between markers 120055 and 120206 in 63 progeny. This region has 69 genes, as predicted by both GreenGenie2 and JGI.

Illumina WGS of NG6 produced 27 million 36-bp single-end reads and sequencing of NG30 produced 200 million 101-bp paired-end reads. Each collection of reads was aligned onto the *Chlamydomonas* version 4 genome assembly (Merchant *et al.* 2007) to quantify coverage of the GreenGenie2 (Kwan *et al.* 2009) predicted exomes in each strain (see the Materials and Methods). For NG6, 92% of predicted exons showed at least fivefold coverage, and for NG30, 95% of predicted exons had at least fivefold coverage. Approximately 18% of the reads could not be aligned onto the JGI version 4 genome assembly in both cases, which may corresponds with the *Chlamydomonas* genome sequence that remains unresolved. Because determination of the number of nonsynonomous changes requires alignment onto the predicted genes, we can only make an estimate of the number of nonsynonymous changes. We estimate that both of the strains have approximately 7000 nonsynonymous changes.

### Identifying a new *FLA8* allele

Because all of the flagellar and basal body mutants in *Chlamydomonas*, excluding insertional mutants, which have been identified molecularly, are coding or splice-site changes, we concentrated our analysis on nonsynonomous or splice-site changes. For NG6, there is only one nonsynonomous mutation in the 269-kb region; it had 37 reads. It is a T to A transversion that changes a valine to a glutamic acid in the *FLA8* gene at position 4457600 on the chromosome, which encodes amino acid 12. *FLA8* encodes a protein that is one of the two motor subunits for intraflagellar transport (IFT) (Cole *et al.* 1998; Miller *et al.* 2005; Piperno and Mead 1997). Sanger sequencing confirmed the change in the NG6 strain (data not shown). Ten independent diploid strains heterozygous for NG6 and the wild-type allele show a wild-type phenotype, whereas 10 diploid strains heteroallelic for NG6 and the *fla8-1*, a temperature-sensitive flagellar assembly mutant, show the phenotype of the *fla8-1* strain. These complementation results suggest that NG6 is likely to be a *FLA8* allele.

As a second line of evidence that the V_12_E change is responsible for the flagellar phenotype, NG6 cells were mutagenized with ultraviolet light to isolate suppressors/revertants. We identified 10 strains and each was crossed to a wild-type strain (CC-125) to ask whether suppression was linked or unlinked. In 9 of the 10 strains, the second event is tightly linked in 28-37 tetrads for each strain. A total of 450 bps around the V_12_E change in *FLA8* were sequenced for each of these nine reverted strains. Five strains were true revertants that change the mutant GAA codon back to GTA. Four different codons were observed among the remaining strains and they are pseudorevertants. They included glycine (2), isoleucine (1), and leucine (1) codons. One strain (REV3) had no change in the sequenced region, and the change may have resided outside of the sequenced region (Table 1). Thus, it appears to be highly likely that the NG6 encodes the kinesin-2 subunit, FLA8.

**Table 1:** Intragenic revertants of NG6 (*fla8-3*)

Name	Codon	Amino acid
Wild-type	GTA	V
Parent: NG6	GAA	E
REV1	GTA	V
REV4	GTA	V
REV5	GTA	V
REV7	GTA	V
REV11	GTA	V
REV2	ATA	I
REV3	GAA	E
REV6	GGA	G
REV9	GGA	G
REV10	TTA	L

**Figure 1:**
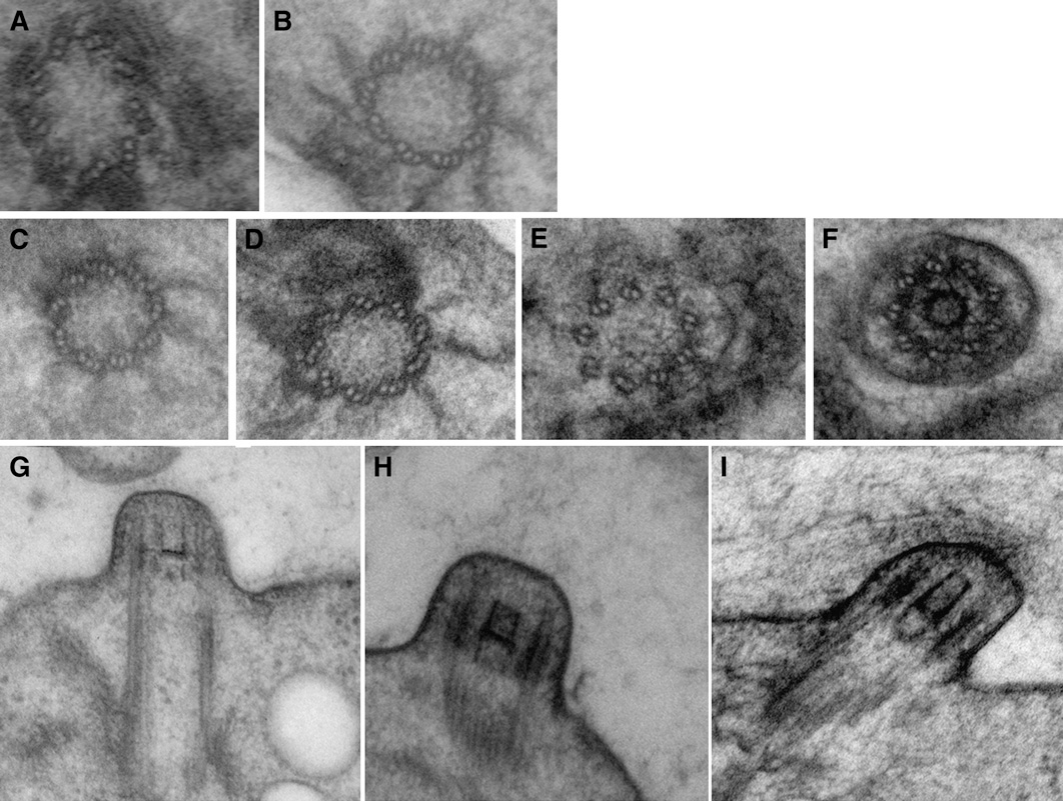
Electron microscopy of basal bodies from NG6 (*fla8-3*) and NG30 (*ift80-1*). (A, B) Cross-sectional images from NG6 showing closed tubules and no hooked tubules. (C−F) Cross-sectional images of NG30 showing normal transition zones. (G) Longitudinal image of NG6 without a cap at the distal end of the transition zone. (H, I) Longitudinal images of NG30 with cap at the distal end of the transition zone. This cap is not observed in wild-type cells or other mutants.

Because the electron micrographs of McVittie (1972) suggested that the B-tubules fail to close in this mutant and there is no evidence that kinesin motors affect B-tubule closure in any organism, we re-examined the cells by electron microscopy after high-pressure freezing and freeze substitution (O’Toole *et al.* 2003). Because none of the NG6 cells had flagella, basal bodies were examined. After examining the basal bodies from 22 cells, no basal bodies with open B or C tubules were observed (Figure 1, A and B), and the transition fibers appeared unaffected in cross-sectional views (Figure 1B). Longitudinal views suggest the transition zone and flagellar tunnel also were normal (Figure 1G).

### Identifying an *IFT80* allele

The NG30 genome has a total of 38,177 changes relative to the reference genome. The 458-kb region that contains the NG30 mutation had six nonsynonymous changes in six different genes that have from 41 to 248 reads each (Table 2). One of the genes, gg2_c3_t1112, encodes a protein with well-conserved homologs; it encodes IFT80, which is a protein in Complex B of the IFT complex (Cole *et al.* 1998; Piperno and Mead 1997). Recently, mutations in *IFT80* have been found in patients with Jeune asphyxiating thoracic dystrophy and in mice (Beales *et al.* 2007; Rix *et al.* 2011). These patients have a narrow thorax and short limbs together with respiratory and renal defects that are often observed in other ciliopathies. Sanger sequencing confirmed the change in the NG30 strain (data not shown). To validate the gene responsible for the phenotype, we rescued the nonswimming phenotype with a DNA fragment from BAC 15M19 that had been cut with restriction enzymes to produce an 8.2-kb fragment with only the *IFT80* gene (*Avr*II and *Cla*I). Restoration of flagellar assembly and swimming was observed in 10 independent transformants transformed with the purified 8.2-kb fragment, and no rescue was observed after transformation with an adjacent fragment of 5.6 kb obtained by *Aat*II and *Cla*I digestion or with two adjacent BACs (39O2 and 23L15) when five times the DNA concentration used for the 8.2 kb fragment was electroporated into cells.

**Table 2:** NG30 nonsynonymous mutations in the 458 kb region

GreenGenie2 name	Version 4 position	Protein ID (JGI)	Amino acid change	Nucleotide change	Number of reads
c3_t1077	6358278	377898	S->L	TCG->TTG	46
c3_t1102	6547875	205509	Q->X*^a^*	CAA->TAA	157
c3_t1112	6608262	24171	W->X*^a^*	TGG->TAG	248
c3_t1127	6689834	147508	A->G	GCG->GGG	41
c3_t1128	6692716	293428	A->P	GCC->CCC	63
c3_t1132	6711089	418195	V->G	GTG->GGG	103

a X = Nonesense codon.

Meiotic progeny from five of the swimming transformants were crossed to the CC-124 strain; the analysis showed the rescue is unlinked to NG30. Ten pair-wise crosses among the five transformants showed the rescuing insertions occurred at five unlinked locations in the genome in 15-22 tetrads. Although we did not attempt to rescue the mutant phenotype with a DNA fragment that covers the other five genes with a nonsynonomous change in Table 2, we feel confident that the causative mutation is in the *IFT80* gene because a fragment with only the *IFT80* gene (Table 2) rescues the aflagellate, nonswimming phenotype.

Electron microscopy of *bld1-1*, which is a mutant in the IFT52 subunit in Complex B of IFT, did not show abnormal transition zone material (Deane *et al.* 2001) as was observed for NG30 by McVittie (1972). Therefore, we again used electron microscopy to examine the NG30 cells. We failed to find abnormal extended transition zone material in the NG30 mutant (Figure 1, C−F, H−I). However, we observed a unique structure at the distal end of the transition zone in NG30 cells in longitudinal sections. A bar or cap is observed over the distal end of the transition zone (Figure 1, H and I). This structure has not been observed in wild-type or other mutant cells (O’Toole *et al.* 2003; Preble *et al.* 2001; Figure 1G).

Because we used NG6 and NG30 strains that had been backcrossed four times for both sequencing and electron microscopy, it is possible that the starting strains had multiple mutations that independently produced the aflagellate phenotype and the transition zone phenotype and that the two phenotypes were genetically separable. We went back to the original NG6 and NG30 strains obtained from the *Chlamydomonas* Center and carefully examined progeny from a new backcross. Although these crosses only gave approximately 15% viability, we found no evidence of other mutations that affect any aspect of flagellar assembly or function after examining motility, phototaxis, and mating by standard assays.

### Failure to find *ac17*

The *ac17* mutant requires the addition of acetate to the medium (Levine and Goodenough 1970), and it maps to chromosome 3 near the centromere between the phenotypic markers *ac208* and *y7* (Harris 2009) and between dCAPs markers 953 and 751, which span 676 kb of sequence (Table S1) based on 120 progeny. This region contains 35 genes predicted by GreenGenie2 and 78 genes predicted by JGI. The *ac17* strain (CC-530) was sequenced and 180 million 101-bp paired end reads were obtained. 95% coverage of the predicted exons with at least fivefold coverage was obtained. This strain has a total of 40,463 changes. Unlike the regions where NG6 and NG30 map, this interval contains highly repetitive DNA sequences and lacks an intact scaffold (Merchant *et al.* 2007). We found no nonsynonymous or splice-site changes in the mutant sequence compared with the GreenGenie or JGI predictions in this region. To gain on estimate of the number of genes that might be missing in these poorly assembled regions, we used CEGMA (Cantarel *et al.* 2008; Parra *et al.* 2007), which uses 248 well-conserved genes as a measure of the completeness of a genome assembly. By BLAST against the *Chlamydomonas* genome, we found that 238 of the 248 core conserved genes were present, which provides an estimate that only 4% of the coding genes are missing from the genome assembly.

### Polymorphisms in the mating-type locus

The mating-type locus of *Chlamydomonas* has two alleles, *mt* plus and *mt* minus. Ferris and Goodenough (1994) showed that the two alleles on chromosome 6 are embedded in a region of DNA that contain translocations, inversions, duplications, and deletions, which creates a region that is suppressed for recombination. Each locus contains mating-type specific loci (*MID1*, *MTD1* only in the *mt* minus locus, and *FUS1 MTA1* only in the *mt* plus locus). There are also many housekeeping genes that are contained within this scrambled region (*e.g.* *NIC7*, *THI10*, *AC29*) (Ferris 1995; Lin *et al.* 2010). The locus is estimated to be 640 kb long (Ferris and Goodenough 1994), which was revised to 366 kb by comparison to the *Volvox* mating-type locus (Ferris *et al.* 2010). Because it is believed that this region has failed to recombine over a fairly long time period, it seems likely that there will be increased numbers of DNA changes in the mating-type locus compared with other loci and chromosomes (Charlesworth and Charlesworth 2000; Whittle and Johannesson 2011).

**Figure 2:**
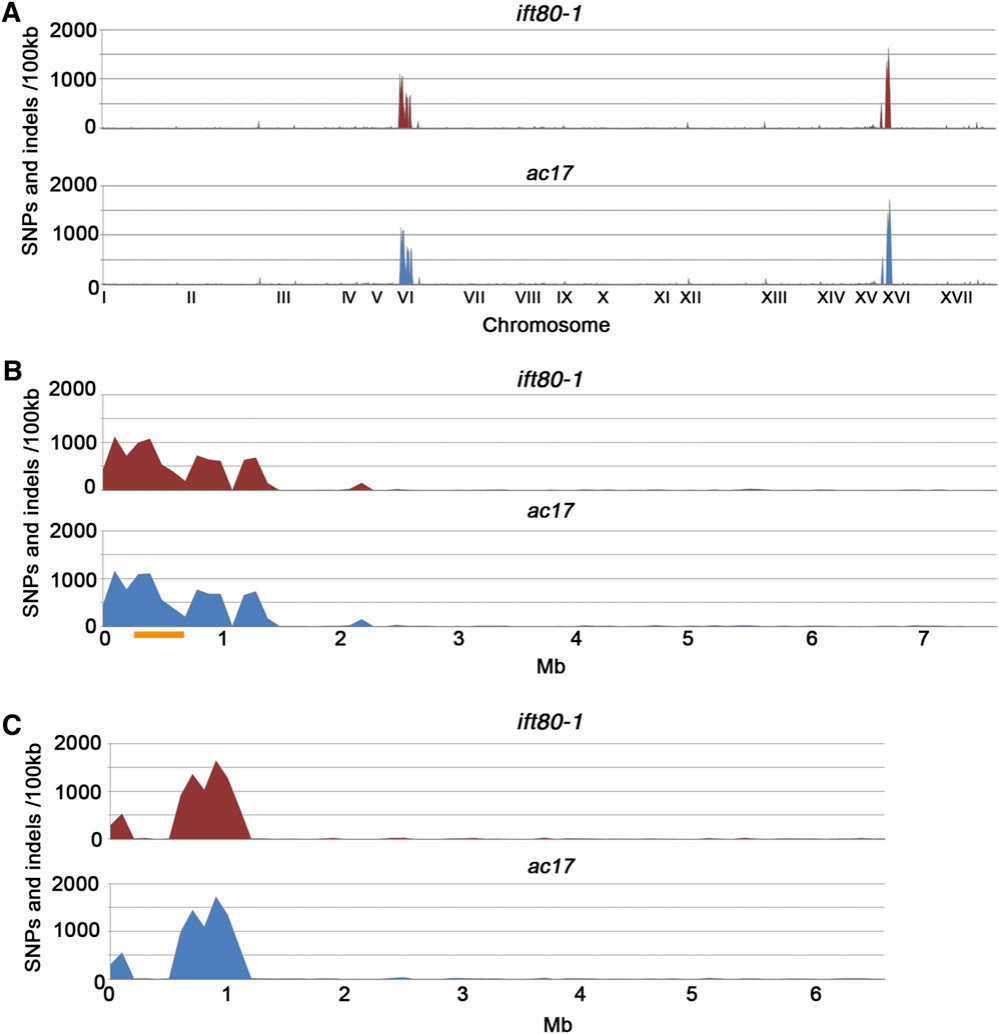
Distribution of changes across the genome by chromosome. (A) Plots of the number of changes (y-axis) in *ift80-1* and *ac17* strains relative to the JGI reference genome. The x-axis shows the 17 chromosomes with their proportional length. (B) Expansion of the plot in panel A for the number of changes on chromosome 6 for *ift80-1* and *ac17*. (C) Expansion of the plot in panel for the number of changes on chromosome 16 for *ift80-1* and *ac17*.

We compared the sequence from the two strains sequenced with 101bp paired end reads (*ift80-1/*NG30 and *ac17*) to the JGI sequence on each chromosome. The frequency of SNPs and indels along the 17 chromosomes is plotted in Figure 2A. Surprisingly, both chromosomes 6 and 16 have regions with elevated numbers of changes relative to the reference genome in both strains. A total of 23% of the changes in the *ift80-1*/NG30 and *ac17* strain occur over 1.5 Mb of chromosome 6, which is ∼1.25% of the total genome (Figure 2B; Table 3). Ferris *et al.* (2010) place the mating-type locus between 432 kb and 798 kb on the JGI map, which we have indicated as a orange bar in Figure 2B. Only 5% of our changes occur in this region (Table 3). They compared the 65 genes in the mating-type region of *Chlamydomonas* and *Volvox* and observed dS and dN values between 0 and 0.09. We analyzed 10 genes from chromosome 6 and matched them by function to 10 genes on other chromosomes (Table 4). One of the 10 genes (*IFT139*) falls outside our region of elevated numbers of changes, and it has no changes. Eight of the remaining nine genes had nonsynonymous changes with frequencies from 0.0015 to 0.02, which include two genes (*GCSH1* and *UTP1*) in region defined as the mating-type locus (Ferris *et al.* 2010). The value in Table 4 are similar to the comparisons made with *Volvox*. The ninth gene (*FA1*) has an indel in the coding region. With the exceptions of *FA1* and *IFT139*, which have no synonymous changes, the frequency of synonymous changes ranged from 0.0005 to 0.035, findings that are similar to the values of Ferris *et al.* (2010).

**Table 3:** Variation on chromosomes 6 and 16

Chromosome	*ift80-1*/NG30		*ac17*	
	Number	Percent	Number	Percent
Chromosome 6 (432-798 kb)	1882	4.93	1911	4.72
Chromosome 6 1 bp-1.5 Mb	9013	23.61	9464	23.93
Chromosome 16 (1 bp-1.2 Mb)	7848	20.56	8254	20.40
Total changes	38177	—	40463	—

**Table 4:** Synonymous (S) and nonsynonymous (NS) changes in mating-type linked and unlinked genes

Gene name	Position (kb)	No. amino acids (fraction of NS and S changes)	NS NG30/*ac17*	S NG30/*ac17*
Mating-type linked genes and their map position in reference (V4)			
*NIC7*	(342432-344301)	633	1/1	20/22
		0.0015 (NS)		
		0.031–0.035 (S)		
*ALB3.1*	(387003-390870)	495	3/3	5/6
		0.006 (NS)		
		0.01–0.012 (S)		
*GCSH*	(612078-614002)	159	2/2	2/2
		0.0125 (NS)		
		0.0125 (S)		
*UTP1*	(789721-794290)	996	9/10	12/13
		0.009–0.10 (NS)		
		0.012–0.013 (S)		
*SAD1*	(883075-898052)	4027	14/16	19/20
		0.003–0.004 (NS)		
		0.0047–0.005 (S)		
*MAT3*	(939371-945298)	1213	4/4	16/16
		0.0049 (NS)		
		0.013 (S)		
*THI10*	(931565-934443)	277	3/3	4/6
		0.010 (NS)		
		0.014–0.020 (S)		
*FA1**^a^*	(1199816-1210569)	1787	0/0	0/0
		0		
*LEU2*	(1363805-1371222)	444	2/2	5/5
		(0.004) (NS)		
		(0.011) (S)		
*IFT139*	(2451412-2466548)	1347	0/0	0/0
		0		
Genes in corresponding pathways on other chromosomes			
*NIC15*	chromosome 12	669	0/0	0/0
*FA2*	chromosome 7	618	0/0	0/0
*ALB3.2*	chromosome 12	422	0/0	0/0
*OGD2*	chromosome 7	450	0/0	0/0
*WDR36*	chromosome 10	730	0/0	0/0
*SAG1*	chromosome 8	3409	0/0	0/0
*DP1*	chromosome 7	646	0/0	0/0
*THI4*	chromosome 4	357	0/0	0/0
*IDH3*	chromosome 2	483	0/0	0/0
*IFT144*	chromosome 13	1384	0/0	0/0

a The *FA1* gene has an insertion of a single base (G) at position 1208943 in the *ac17* mutant strain. The wild-type protein is 1787 aa long; the mutant protein would be 1742 aa. Their amino acid sequences differ after amino acid position 1686. No other indel were observed.

In both strains, chromosome 16 has a region of increased number of changes. Approximately 20% of the genomic changes over 1.2 Mb of the left arm, and only 2.6% changes over the remaining 5.3 Mb of chromosome 16 in both strains (Table 3).

## Discussion

Recently many workers in *Chlamydomonas* have relied on insertional mutants for identifying genes of interest because of the greater ease of identifying the mutated genes using the inserted DNA as a tag (Pazour and Witman 2000). This method is used despite the fact that many insertional mutants remove large regions of DNA and multiple genes (Myster *et al.* 1997; Nakazawa *et al.* 2007) and that many chemical and light induced mutations exist that may provide additional information about the function of genes (Harris 2009). To ask whether WGS is a viable approach for *Chlamydomonas*, we started with three mutants that had been mapped to relatively short regions ranging from 275 to 676 kb. The first finding is that each strain has >38,000 changes compared with the reference genome (Table 3). Because these strains were backcrossed four times, it is not known whether these changes came from long-term culture and selection (>40 years), from the original mutagenesis used to generate these strains, or from differences between the laboratory strain and the reference genome. To address this, we have preliminary sequencing data that suggest that CC-125 has approximately 24,000 changes relative to the reference genome. Approximately one-quarter of these are present in the *ift80-1*/NG30 and *ac17* strains (H. Lin and S. Dutcher, unpublished data), which suggests that there are differences among the isolates commonly used in the laboratory. The differences may arise from long-term storage and/or the mutagenesis. Sequencing of more mutants and strains will be needed to address these questions.

The second finding is that two of the mutants have a small number of coding variants in the regions of interest. To validate the sequencing changes in the genes of interest, we used intragenic revertants for NG6 (Huang *et al.* 1981; Miller *et al.* 2005) and transformation rescue for NG30 (Iomini *et al.* 2009a). NG6 is a mutation in *FLA8*, and we renamed it *fla8-3*. NG30 carries a nonsense mutation in *IFT80*, and we named it *ift80-1*. Both of these regions surrounding these genes appear complete in the reference genome (Merchant *et al.* 2007) in contrast to the region, where *AC17* maps. There were no candidates for *AC17* with nonsynonymous changes in our sequence. This region contains many gaps in the assembly, and it is likely that genes of interest are missing. If we had not mapped NG6 and NG30, we would have failed to find the causative changes among the large number of other changes. We are currently trying an approach that uses the polymorphic CC-1952 strain (Gross *et al.* 1988) in which we collect multiple meiotic progeny with the phenotype of interest from a cross. We search for regions that lack heterozygosity; this approach is conceptually similar to one used previously in *C. elegans* (Doitsidou *et al.* 2010).

The third finding is that the recombinationally suppressed regions around the mating-type locus on chromosome 6 show an elevation in the number of changes relative to other regions of the genome (Figure 2; Table 3). As has been suggested previously, this may arise from the lack of pairing between homologs that would allow repair. The mating-type region was defined originally as approximately 640 kb long (Ferris and Goodenough 1994) and was recently revised to be only 366kb (Ferris *et al.* 2010). However, the region with elevated number of changes extends over 1.5 Mbps in the two strains compared with the reference genome (Merchant *et al.* 2007).

To ask whether recombination is affected over the 1.5 Mb, we re-examined data obtained in previous work in which we mapped several genes around the mating-type locus (Iomini *et al.* 2009a). On the basis of the JGI genome assembly the *IFT139* (*FLA17*) and *FUS1* genes are approximately 1.9Mb apart, but they are only 1.4 mu apart genetically. This result suggests that there is significant recombinational suppression. In most regions of the genome, 1 mu corresponds to 102-105 kb (Harris 2009). If there were recombinational suppression only within the 366- to 640-kb mating-type locus, we would expect a map distance of 12 mu from the mating-type to *IFT139* (Ferris *et al.* 2010; Ferris and Goodenough 1994).

It appears that there is a significant repression of recombination throughout this region. However, there may be elevated recombination outside of the interval of elevated SNPs as the genetic distance from *BBS5* to *IFT139* is 5 mu (Iomini *et al.* 2009a). Although these loci map to 2.34 Mb and 2.46 Mb, respectively, in the JGI assembly, our mapping places them in the order *MT-IFT139-BBS5*. If this spacing is correct, then these loci show a fivefold increase in recombination compared with the genomic average. These data argue that there is recombinational suppression beyond the 366/640 kb interval proposed for the mating-type region and may be accompanied by increased recombination outside the region of elevated SNPs. In the future, the analysis of recombination throughout this region could be easily monitored in several hundred meiotic progeny and provide new insights into generation of meiotic recombinants.

The high degree of SNPs on chromosome 16 is unexpected. One explanation is that this region also contains inversions and other rearrangements that suppress recombination, or sites that prevent repair. There is insufficient genetic data with known loci to make further conclusions (Harris 2009).

The ultrastructural phenotype of basal bodies in *ift80-1* is novel. The presence of the cap on the transition zone has not been observed previously. It may be an intermediate in flagellar assembly that requires IFT80 for its removal and thus is not observed in wild-type strains, if it is a rapid step. Because the defect is recessive, we do not think it is due to the build-up of mutant IFT80 protein. If this structure is an intermediate, then the analysis of wild-type strains as they begin flagellar assembly may show a similar structure. Examination of other IFT mutants (Iomini *et al.* 2009a) for the presence of this cap at the distal end of the basal body may identify a subset of the IFT complex that is involved in its removal, which may elucidate another function of the IFT complex.

In summary, WGS appears to be a viable approach for identifying causative mutations in *Chlamydomonas* when it is combined with limited mapping. In addition, it has provided new insights into regions of the genome that may show altered recombination and repair.

## Supplementary Material

Supporting Information
