# Supplementary material for: Whole-Genome Sequencing to Identify Mutants and Polymorphisms in Chlamydomonas reinhardtii
Source: G3 (Bethesda). 2012 Jan 1;2(1):15–22. doi: 10.1534/g3.111.000919 (PMC3276182; doi:10.1534/g3.111.000919)
Supplement: Supporting Information [file supp_2_1_15__index.html]

Supporting Information 

# Whole-Genome Sequencing to Identify Mutants and Polymorphisms in *Chlamydomonas reinhardtii*

## Supporting Information for Dutcher *et al.*, 2012

**Files in this Data Supplement:**

- Table S1
