# Supplementary material for: Whole-Genome Sequencing to Identify Mutants and Polymorphisms in Chlamydomonas reinhardtii
Source: G3 (Bethesda). 2012 Jan 1;2(1):15–22. doi: 10.1534/g3.111.000919 (PMC3276182; doi:10.1534/g3.111.000919)
Supplement: Supporting Information [file supp_2.1.15_TableS1.pdf]

Table S1 Primers for mapping NG6, NG30 and *ac17*

Marker	Sequence 5' to 3'	Sequence 5' to 3'	Map Position JGI v4	Temperature	Restriction Enzyme
Chromosome 12					
160081	TTG GAG TTT GAG CTG CAA TG	CCA GCC CCA TGA GAA CTA AA	3884888-3885293	53°C	*Hae*III
160178	AGC TGG ACC TGG TTC AAA TG	ATG CCG TAC TCC CAC CTG	4089949-4090378	53°C	Size difference
160043	GCG AGA TCA TCA CGG ACT TC	GCA TGC GCT TGC ATA CTA TC	4234642-4234935	53°C	*Hae*III
Bug24	GAC ATC GAA TCT TCC CAA CG	AGT CAA GCA CTT GCC GAG AT	4289729-4290144	53°C	*Msp*I
160152	CTG AAA GAG CGG ATT GTG GT	CTG CAT CGT GTC GTA AAT GG	4321000-4321370	54°C	*Msp*I
160146	TGG TCA CGT AGC TGC ATA GC	CCC TGA CAA ACA CCA CAC AC	4335619-4388402	52°C	*Rsa*I
Fla8	TAT CAA GCC CAC GGG AGT AG	CGG ATG TGT TAC GAG TGT CG	4462079-4462321	53°C	*Rsa*I
160197	AGG AAG CCC GTG TGT GTA T	CAT AGT ACC CGG ACG CTT GT	4485972-4486542	53°C	Size difference
160085	CCT CTC CTT	TGG GTG GGC	4543180-	53°C	*Mnl*I

	TTG GAA TGA CG	ACC TAA TAC AC	4543569		
269006	GGC AGG AGG GAA GTG GAG	CCC ACC TCA ACA CAC ACA CA	4605260 - 4605589	55°C	Size difference
380038	ATC ATC TGT GTG CCC ATG C	GCC CGT GTA TCC TGT CAA TC	4964322-4964798	53°C	Size difference
LC3	TGC ACA ACA CAA CAA GCA GA	ACA GGC GAG GAC ACA GCT AC	5150218 - 5150592	53°C	*Hae*III
NPHP4	Cca tgc tgg gtg aa tga t	GGG TCC ACC TCG TTG ATC T	5408391-5408949	53°C	*Msp*I
**Chromosome 3**					
DMAT	GGA CAT TCG TGT GGA GTG AA	AGG AAA CGC AGT CAA GGG TA	2766483-2766784	50°C	Size difference
TUA1	GGC CAT GAG TTG CTT CTT TC	AGG AAA CGC AGT CAA GGG TA	5007715-5008194	53°C	*Msp*I
PHOT	TGC AGT TTT GCA GTT TGG AG	CTG CCG TCC ATG TTC CTT AT	5939889-5940314	51°C	*Dde*I
120055	GTG ATG GGC TAC CAG ACG TT	GCT GTC CAG GTT CTT CAG GA	6274237-6274567	53°C	*Hae*III
IFT80	TTG TGC AAG TGT CGT GTC AA	GCT GCT GTT AAT TCG CTG TG	6607725-6608200	53°C	*AlwN*I
120206	Acg cgc tgt gta	TCT GCA GCG	6731486-	51°C	*Dde*I

	acg agt c	TGT TGT TAC AG	6731869		
953	GTG TGC GTG TCA GTA TGA GCT GG	CGC AGG ACA GTG TAC CTA CCG TTT	6840208-6840425	53°C	Size difference
120019	GCA CAT GCC ATC ATA TCA GC	AGC GCT ACA CCA GAA CGA GT	7043084-7043541	50°C	*Msp*I
751	AAA CCC ACA TAA CCC ACA GG	GCC ATG GTG CAG TGT AGA GA	7516918-7517293	53°C	Size difference

* Primers 160146 produce a PCR product of 290 bps although Version 4 of the Chlamydomonas genome assembly places them 48.78 kb apart. This marker is linked to NG6. This suggests a small misassembly in Version 4.
